# The Gender Gap in Life Expectancy in Urban and Rural China, 2013–2018

**DOI:** 10.3389/fpubh.2022.749238

**Published:** 2022-02-11

**Authors:** Jinjing Wu, Samir KC, Marc Luy

**Affiliations:** ^1^Asian Demographic Research Institute, Shanghai University, Shanghai, China; ^2^International Institute for Applied Systems Analysis (IIASA), Wittgenstein Centre for Demography and Global Human Capital (IIASA, OeAW, University of Vienna), Laxenburg, Austria; ^3^Vienna Institute of Demography (OeAW), Wittgenstein Centre for Demography and Global Human Capital (IIASA, OeAW, University of Vienna), Vienna, Austria

**Keywords:** gender gap, life expectancy, cause of death, urban-rural difference, China

## Abstract

**Background:**

Gender differences in mortality are embedded within mortality transitions. Rural residents generally lag behind their urban counterparts in the transitions. The study objective is to identify major causes of death that drive gender differences in mortality in urban and rural China.

**Methods:**

We use age-, gender-, urban-rural- and cause-specific mortality data (2013–2018) derived from the national mortality surveillance system that covered about 24% of the Chinese population. We apply Arriaga's method to decompose age- and cause-specific contributions to the gender gap in life expectancy at birth. Analyses are stratified by urban-rural residence.

**Results:**

Women had a higher life expectancy at birth than men in both urban and rural areas. Cancers, cardiovascular disease, external causes, and respiratory disease accounted for more than 90% of the gender gap in both areas during 2013–2018. In urban areas, the gender gap decreased from 5.17 years in 2013–2015 to 4.98 years in 2016–2018. In rural areas, the gender gap stayed rather constant (2013–2015: 5.68 years; 2016–2018: 5.65 years). Traffic accidents, among external causes, contributed the most to decreasing the gender gap (urban: −0.07 years; rural: −0.10 years), especially in the 0–44 age group. However, the decrease in the gender gap was counteracted by an increase in the gender gap attributable to ischemic heart disease (urban: +0.05 years; rural: +0.08 years) and lung cancer (urban: +0.02 years; rural: +0.05 years) in older age groups. The gender gap attributable either to cerebrovascular disease or to chronic lower respiratory disease decreased in urban areas but increased in rural areas.

**Conclusions:**

The urban-rural variations in the cause-specific contributions to the gender gap in China suggest the necessity of implementing urban-rural-specific interventions to improve population health and health equity.

## Introduction

Women generally live longer than men due to biological, social-structural, psychosocial, and behavioral factors (1), except in a few of the world's poorest countries where men still outlive women (2). However, the gender gap in life expectancy at birth (hereafter, “the gender gap”), embedded within mortality transitions, varies over place and time (2). Decomposing the gender gap and its variations by cause of death will help to identify which causes of death need closer monitoring and reveal opportunities to improve gender equity in longevity.

Evidence from Western developed countries shows that cardiovascular disease (CVD), one of the most common non-communicable diseases (NCD), contributes a substantial portion of the gender gap (3, 4). Most Western developed societies have witnessed a decrease in the gender gap since the 1980s (3–7), which parallels a steady decrease in the CVD mortality in the West (8, 9).

Cigarette smoking is a prominent risk factor for developing NCD including CVD, lung cancer, and chronic lower respiratory disease (10). Decreased smoking among men and increased smoking among women explain a substantial portion of the decrease in the gender gap in Western developed countries (3, 11–13). However, the extent to which the decrease in the gender gap can be explained by the trends of women's and men's smoking behaviors in the course of the so-called “smoking epidemic” differs between countries (14).

In addition to NCD, traffic accidents are also a major cause of death responsible for gender differences in mortality (3). In Western industrialized countries, traffic accident mortality rates increased in the post-World War II period (1945–1970) but decreased thereafter (15). Men's greater improvements in mortality from traffic accidents over recent decades have contributed to a notable portion of the decreasing gender gap in these countries (3). Most studies on the gender gap are conducted in industrialized societies, especially in the West (3–7), providing only a partial picture of the evolving gender gap along with mortality transitions. Given varying economic development levels, health system performance, and cultural backgrounds, the evolution of the gender gap and cause-specific contributions in developing countries may differ from those of developed countries.

China, one of the world's fastest-growing economies, has experienced a rapidly growing burden of NCD and traffic accidents, accompanying the rising economic prosperity (16, 17). The rapidly growing burden of NCD in China is not only accelerated by an emerging obesity epidemic due to a nutrition transition toward increased energy intake and decreased physical activity (18); it is also driven by a rapid increase in the number of male deaths from smoking as a consequence of a high male smoking rate (10, 16). The male excess mortality may be increased along with the growing burden of NCD and traffic accidents as men are more likely to engage in risky behaviors including unhealthy eating, smoking, and unsafe driving than women (1, 2). However, evidence on the contributions of NCD (e.g., CVD, lung cancer, chronic lower respiratory disease) and traffic accidents to the gender gap remains limited and inconclusive in the context of China (19, 20).

Due to an urban-rural dual system established in the early 1950s, urban people generally have higher income and better health care access (21, 22). Moreover, the smoking, obesity, and NCD epidemics started in urban areas and then spread into rural areas (23–25). The urban-rural heterogeneities provide a unique opportunity to examine potential drivers (e.g., health care access, smoking, obesity) of variations in the gender gap and inform urban-rural-specific interventions that aim to improve population health and health equity. However, to the best of our knowledge, the urban-rural variations in the cause-specific contributions to the gender gap have not been addressed in the literature so far.

The main objectives of this study include (1) estimating the gender gap in China during 2013–2018 with data from the Chinese national mortality surveillance system, (2) investigating how the gender gap changed from 2013–2015 to 2016–2018, (3) clarifying the cause-specific contributions to the gender gap, with a focus on CVD, lung cancer, chronic lower respiratory disease, and traffic accidents, and (4) examining whether the cause-specific contributions varied by urban-rural residence.

## Materials and Methods

### Data, Sample and Variables

The Chinese government established a nationally and provincially representative mortality surveillance system in 2013 by combining the vital registration system of the National Health Commission of China that was built in the 1950s and the disease surveillance points system of the Chinese Center for Disease Control and Prevention that was built in 1978 (26). In 2013, the national mortality surveillance system comprised 605 surveillance points in total, with each point representing an entire district or county (26). Out of the 605 surveillance points, 158 and 113 points were retained from the disease surveillance points system and the vital registration system, respectively, while the rest were newly added in 2013 (26). The system covered 31 provinces and more than 0.3 billion people, making up 24% of the Chinese population (26). To ensure representativeness at the provincial level, each county or district is selected using an iterative method involving multistage stratification that considers the sociodemographic characteristics of the population (26). More details can be found in (26).

The national project team excludes the surveillance points with unreasonably low mortality from the estimation of national mortality rates. Among the surveillance points established before 2013, the points having a mortality rate of below 4.5‰ are excluded; among the points newly added in 2013, the points having a mortality rate of below 5.0‰ are excluded (27). Thus, the mortality data reported come from 432, 491, 491, 499, 509, and 512 out of 605 mortality surveillance points in 2013, 2014, 2015, 2016, 2017, and 2018, respectively (27).

From the China Health Statistics Yearbooks, we obtain the age-, gender-, urban-rural- and cause-specific mortality rates derived from the national mortality surveillance system (28). We do not include the mortality data collected before 2013 in our analyses as the data is not nationally representative and thus not comparable to the data collected since 2013. The China Health Statistics Yearbooks do not report the number of respondents and deaths in the national mortality surveillance system. Therefore, it is not possible to provide uncertainty estimates.

#### Cause of Death

The causes of death are classified by the tenth revision of the International Statistical Classification of Diseases and Related Health Problems (ICD−10). Consistent with (19), we include seven chapters of ICD−10 (i.e., cancer; cardiovascular disease; external causes; respiratory disease; digestive disease; infectious disease; endocrine, nutritional, and metabolic disease) and thirty-two subchapters into our analyses. These chapters and subchapters cover the most common causes of death in China (29) and are most relevant to the gender gap (19, 20). In addition, we include two categories that cover other causes and unspecified causes, respectively.

#### Urban-Rural Residence

The sampling unit of the national mortality surveillance system is the district or county (26). In China, districts and counties are at the same administrative level under prefecture-level cities; there are urban and suburban districts, while counties cover the majority of rural areas (30). To be consistent with prior studies (31), we classify districts as urban areas and counties as rural areas.

#### Life Expectancy at Birth

Life expectancy at birth is one of the most widely used summary measures of mortality rates across all age groups observed at a specific point of time (32). To facilitate a comparison of our results with that of previous studies (19, 20, 33, 34), we estimated life expectancy at birth by gender. It can be interpreted as the average number of years that a newborn baby is expected to live, with the assumption that the observed age-specific mortality rates remain unchanged in the future (32).

### Statistical Analyses

#### Life Table Techniques

Life tables by gender and urban-rural residence are constructed using all-cause mortality rates. We use standard life table techniques to estimate the life expectancy at birth by gender and urban-rural residence (35). We use age categories that were 0, 1–4, 5-year age groups starting with 5–9 years up until 80–84, and the last open age interval 85+. More details about life table construction can be found in (34).

#### Arriaga's Decomposition Method

Arriaga's decomposition method has been widely used to decompose differences in life expectancy between men and women by age and cause (19, 20, 33, 34). More details about the development of decomposition methods can be found in (36) and (37). To facilitate a comparison of our estimates to the findings of previous studies, we use Arriaga's method to decompose the gender gap by age and further by cause of death (38, 39).

A gender difference in mortality in all age groups except for the oldest age group has three different effects on total life expectancy: direct, indirect, and interaction effects (38). The gender difference in mortality in the oldest age group only has a direct effect on total life expectancy.

The gender difference in life expectancy attributable to the age group x to x+n is estimated as follows:


(1) $$\begin{array}{l} \Delta_{x}=[\frac{l_{x}^{Men}}{l_{0}^{Men}}\times (\frac{L_{x}^{Women}}{l_{x}^{Women}}-\frac{L_{x}^{Men}}{l_{x}^{Men}})]+[\frac{T_{x+n}^{Women}}{l_{x+n}^{Women}}\\ \;\times \frac{\frac{l_{x}^{Men}l_{x+n}^{Women}}{l_{x}^{Women}}-l_{x+n}^{Men}}{l_{0}^{Men}}] \end{array}$$

We assume that women have a lower mortality rate than men between ages x and x+n. In formula (1), _Δ*x*_ is the contribution of the age group x to x+n to the gender difference in life expectancy at birth. $l_{0}^{Men}$ is the number of men surviving to age 0 (i.e., 100,000). $l_{x}^{Men}$ and $l_{x}^{Women}$ are the numbers of people surviving to the exact age x of 100,000 people in men and women, respectively. $L_{x}^{Men}$ and $L_{x}^{Women}$ represent the number of person-years lived between ages x and x+n in men and women, respectively. $l_{x+n}^{Women}$ is the number of women surviving to the exact age x+n. $T_{x+n}^{Women}$ is the number of person-years lived from age x+n to the oldest age group in women.

The first term on the right-hand side of formula (1) captures the direct effect. The direct effect measures the gender difference in the number of person-years lived between ages x and x+n due to women's lower mortality rate in this age group within the age interval.

The second term on the right-hand side of formula (1) corresponds to the sum of the indirect and interaction effects.

There is an indirect effect resulting from additional female survivors at the end of the age interval, x+n, because of women's lower mortality rate between ages x and x+n. The additional female survivors at the age x+n will pass through successive age groups and then result in an additional number of person-years lived, contributing indirectly to the overall gender gap (38).

Both the direct and indirect effects only consider the gender difference in mortality between ages x and x+n. However, gender differences in mortality exist in almost every age group. The additional female survivors at the age x+n will be exposed to lower (or higher) mortality rates than their male counterparts across successive age groups after the age x+n. The interaction effect results from the combination of the additional female survivors at the age x+n due to women's lower mortality rate between ages x and x+n and the lower (or higher mortality rates at older age groups (38).

We further decompose the gender gap by cause of death, with the assumption that the contribution of each cause to the gender difference in life expectancy at a specific age group is proportional to the contribution of each cause to the gender difference in the all-cause mortality rates in the same age group (33). The gender gap attributable to a specific cause between ages x and x+n is estimated as follows:


(2) $$\begin{array}{r} \Delta_{x}^{i}{=}_{x}\times \frac{p_{x}^{i,Women}-p_{x}^{i,Men}}{r_{x}^{Women}-r_{x}^{Men}} \end{array}$$

In formula 2, $\Delta_{x}^{i}$ is the contribution of the cause i to the gender gap between ages x and x+n. $r_{x}^{Women}$ and $r_{x}^{Men}$ represent women's and men's mortality rates between ages x and x+n, respectively. $p_{x}^{i,Women}$, and $p_{x}^{i,Men}$ are women and men's mortality rates from cause i between ages x and x+n, respectively.

To avoid the strong fluctuation in the gender gap in each year, we estimate the cause-specific contributions to the gender gap for the whole period of 2013–2018 instead of estimating the contributions for each year. To estimate the change in the cause-specific contributions to the gender gap, we compare the cause-specific contributions to the gender gap during 2016–2018 with the contributions during 2013–2015. As the number of respondents and deaths in the national mortality surveillance system is not publicly available, we calculate the mean mortality rates over a period by averaging the year-specific mortality rates. As the samples of the national mortality surveillance system in different years are close in size and represent the same study population, averaging the year-specific mortality rates is relatively valid (40).

## Results

### The Gender Gap in Life Expectancy at Birth During 2013–2018

Life expectancy at birth by gender and urban-rural residence is reported in Supplementary Table S1. Table 1 reports the gender gap in life expectancy at birth during 2013–2018 by urban-rural residence and compares the gender gap in 2013–2015 to the gender gap in 2016–2018. The estimates of the gender gap are positive, showing that women had a higher life expectancy at birth than men in both urban and rural areas during 2013–2018. Moreover, our estimates indicate that the gender gap may be larger in rural areas than in urban areas. As shown in Table 1, the gender gap in urban areas decreased from 5.08 years in 2013–2015 to 4.98 years in 2016–2018. The gender gap in rural areas stayed rather constant.

**Table 1:** Gender gap in life expectancy at birth^a^ in 2013–2018, 2013–2015, and 2016–2018 by urban-rural residence.

	**Urban**	**Rural**
	Gender gap in life expectancy at birth, years	
2013–2018	5.08	5.66
2013–2015	5.17	5.68
2016–2018	4.98	5.65
	Changes in gender gap in life expectancy at birth from 2013–2015 to 2016–2018, years	
From 2013–2015 to 2016–2018	−0.19	−0.03

a *The gender gap in life expectancy at birth is equal to women's life expectancy at birth minus men's life expectancy at birth*.

### Age-Specific Contributions to the Gender Gap During 2013–2018

For clarity, Table 2 reports the gender gap in five broad age groups (i.e., the 0–44, 45–59, 60–74, 75–84, and 85+ age groups) during 2013–2018 by urban-rural residence. As shown in Table 2, the 60–84 age group contributed about half of the gender gap in both areas. The 45–59 age group accounted for above 20% of the gender gap and ranked second in contributing to the gender gap. The 0–44 age group may account for a larger proportion of the gender gap in rural areas than in urban areas. More details about the age-specific contributions to the gender gap during 2013–2018 are reported in Supplementary Table S2.

**Table 2:** Age-specific contributions to the gender gap in life expectancy at birth during 2013–2018.

	**Urban**		**Rural**	
	**Years**	**%**	**Years**	**%**
0–44	0.82	16.06	1.19	20.98
45–59	1.19	23.40	1.31	23.06
60–74	1.98	39.02	1.91	33.73
75–84	0.80	15.70	0.94	16.60
85+	0.30	5.81	0.32	5.63
Total	5.08	100.00	5.66	100.00

### Cause-Specific Contributions to the Gender Gap During 2013–2018

Table 3 presents the cause-specific contributions to the gender gap by urban-rural residence during 2013–2018. Cancer, cardiovascular disease, external causes, and respiratory disease accounted for above 92% of the gender gap in both areas. Cerebrovascular disease contributed the most to the gender gap, especially in rural areas (urban: 0.86 years; rural: 1.02 years), followed by lung cancer (urban: 0.69 years; rural: 0.58 years), IHD (urban: 0.57 years; rural: 0.59 years), and chronic lower respiratory disease (urban: 0.43 years; rural: 0.45 years). Liver cancer and stomach cancer accounted for above 0.42 and 0.25 years of the gender gap, respectively. Traffic accidents resulted in a larger gap in rural areas than urban areas (urban: 0.28 years; rural: 0.48 years).

**Table 3:** Cause-specific contributions to the gender gap in life expectancy at birth^a^ during 2013–2018 by urban-rural residence.

	**Urban**		**Rural**	
	**Years**	**%**	**Years**	**%**
**Cancer**	1.71	33.74	1.72	30.37
Malignant tumor	1.71	33.66	1.72	30.31
Nasopharyngeal cancer	0.03	0.53	0.03	0.53
Esophagus cancer	0.20	3.89	0.22	3.96
Stomach cancer	0.25	4.94	0.28	5.03
Colorectal cancer	0.09	1.80	0.06	1.12
Liver cancer	0.42	8.24	0.49	8.62
Lung cancer	0.69	13.68	0.58	10.28
Breast cancer	−0.16	−3.17	−0.12	−2.08
Cervical cancer	−0.09	−1.71	−0.09	−1.65
Bladder cancer	0.04	0.78	0.03	0.54
Leukocythemia	0.03	0.52	0.03	0.49
**Cardiovascular disease**	1.61	31.70	1.81	32.03
Heart disease	0.70	13.75	0.74	13.04
Chronic rheumatic heart disease	−0.01	−0.19	−0.01	−0.16
Hypertensive cardiopathy	0.04	0.88	0.06	1.07
Ischemic heart disease	0.57	11.15	0.59	10.46
Cerebrovascular disease	0.86	16.87	1.02	18.04
Other hypertensive diseases	0.03	0.64	0.04	0.63
**External causes**	0.63	12.51	1.06	18.66
Traffic accidents	0.28	5.55	0.48	8.55
Accidental fall	0.10	1.95	0.14	2.55
Drowning	0.06	1.28	0.11	1.90
Suicide	0.04	0.73	0.06	1.08
**Respiratory disease**	0.60	11.87	0.56	9.88
Pneumonia	0.11	2.18	0.06	0.98
Chronic lower respiratory disease	0.43	8.39	0.45	8.02
**Digestive disease**	0.17	3.30	0.19	3.42
Gastric and duodenal ulcer	0.02	0.45	0.03	0.52
Intestinal obstruction	0.01	0.13	0.01	0.10
Liver disease	0.10	2.06	0.12	2.14
**Infectious disease**	0.11	2.17	0.12	2.20
Tuberculosis	0.03	0.68	0.04	0.72
Hepatitis	0.04	0.87	0.05	0.90
AIDS	0.01	0.28	0.01	0.24
**Endocrine, nutritional & metabolic disease**	0.03	0.67	−0.01	−0.17
Diabetes	0.03	0.57	−0.01	−0.22
**Other causes** ^b^	0.17	3.29	0.16	2.87
**Unspecified causes of death**	0.04	0.77	0.03	0.75
Total	5.08	100.00	5.66	100.00

a *We included seven chapters and thirty-two subchapters of ICD-10 in the estimation of cause-specific contributions to the gender gap. The selection of causes of death is consistent with a prior study (19) to facilitate a comparison between our study and the prior study. Each chapter contains several subchapters. We did not include all subchapters into our analyses. Therefore, the sum of the gender gap attributable to each subchapter within a specific chapter is not necessarily equal to the gender gap attributable to the chapter*.

b *The category of other causes includes 1) diseases of the blood and blood-forming organs and certain disorders involving the immune mechanism, 2) mental, behavioral and neurodevelopmental disorders, 3) diseases of the nervous system, 4) diseases of the musculoskeletal system and connective tissue, 5) diseases of the genitourinary system, pregnancy, childbirth and the puerperium, 6) certain conditions originating in the perinatal period, and 7) congenital malformations, deformations and chromosomal abnormalities*.

### Cause-Specific Contributions to the Change in the Gender Gap From 2013–2015 to 2016–2018

**Figure 1:**
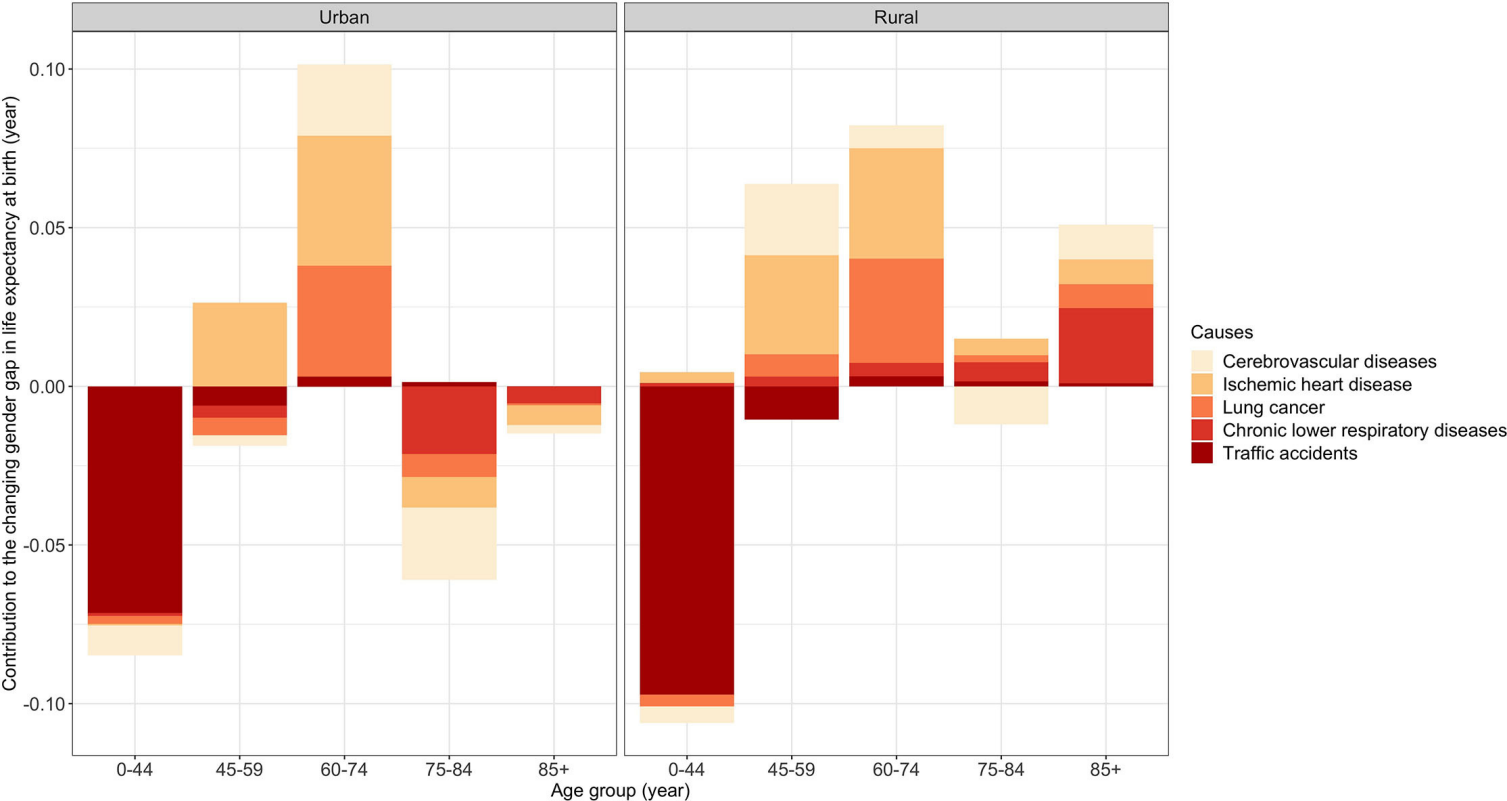
Age- and cause-specific contributions to the changing gender gap in life expectancy at birth from 2013–2015 to 2016–2018 by urban-rural residence.

Figure 1 presents the age- and cause-specific contributions to the change in the gender gap from 2013–2015 to 2016–2018 by urban-rural residence. For clarity, we only report the contributions from cerebrovascular disease, IHD, lung cancer, chronic lower respiratory disease, and traffic accidents in the four broad age groups (i.e., 0–44, 45–59, 60–74, 75–84, and 85+ age groups). More details about the cause-specific contributions to the change in the gender gap are reported in Supplementary Tables S3, S4.

Traffic accidents were the main contributor to decreasing the gender gap in both areas from 2013–2015 to 2016–2018 (urban: −0.07 years; rural: −0.10 years) (Supplementary Tables S3, S4). Figure 1 further shows that the decrease in the gender gap attributable to traffic accidents was mainly concentrated in the 0–44 age group.

IHD (urban: +0.05 years; rural: +0.08 years) was one of the main contributors to the increase in the gender gap in both areas (Supplementary Tables S3, S4). We saw a notable increase in the gender gap attributable to this cause in the 45–74 age groups in both areas (Figure 1). However, in urban areas, the gender gap attributable to this cause decreased in the 75–84 and 85+ age groups (Figure 1). In rural areas, the 0–44, 75–84, and 85+ age groups had a modest increase in the gender gap attributable to this cause (Figure 1).

Lung cancer (urban: +0.02 years; rural: +0.05 years) was the other main contributor to the increase in the gender gap in both areas (Supplementary Tables S3, S4). As shown in Figure 1, in urban areas, the gender gap attributable to this cause had a notable increase in the 60–74 age group. However, it was partly offset by a slight decrease in the gender gap due to this cause in the other age groups except for the 85+ age group. In rural areas, there was a notable increase in the gender gap due to this cause in the 60–74 age group. In contrast to urban areas, this cause resulted in a slight increase in the gender gap in the other age groups except for the 0–44 age group.

The gender gap attributable to cerebrovascular disease decreased in urban areas but increased in rural areas (urban: −0.02 years; rural: +0.02 years) (Supplementary Tables S3, S4). According to Figure 1, although this cause increased the gender gap in the 60–74 age group in urban areas, it decreased the gender gap in the other age groups, especially in the 75–84 age group. In rural areas, it resulted in a slight decrease in the gender gap in the 0–44 and 75–84 age groups, which cannot offset the increase in the gender gap due to this cause in the 45–59, 60–74, and 85+ age groups.

The gender gap due to chronic lower respiratory disease decreased in urban areas but increased in rural areas (Supplementary Tables S3, S4). As shown in Figure 1, in urban areas, the gender gap attributable to this cause decreased in the 45–59 and 75+ age groups and remained stable in the other age groups. However, in rural areas, this cause increased the gender gap in all the age groups, especially the 85+ age group.

According to Supplementary Tables S3, S4, cervical cancer, breast cancer, stomach cancer, and drowning also decreased the gender gap in both urban and rural areas, while colorectal cancer and diabetes increased the gender gap. The gender gap attributable to liver cancer decreased in urban areas but remained stable in rural areas. The gender gap due to accidental falls remained stable in urban areas but increased in rural areas.

## Discussion

This study adds to the literature by assessing the cause-specific contributions to the gender gap over the recent period in China, a newly industrialized country, and identifying the urban-rural differentials in the cause-specific contributions. During 2013–2018, the gender gap was 5.08 years in urban areas and 5.66 years in rural areas. The gender gap decreased from 5.17 years in 2013–2015 to 4.98 years in 2016–2018 in urban areas but remained rather constant in rural areas.

Our results indicate that CVD, cancers, external causes, and respiratory disease were four major categories of causes of death responsible for the gender gap during 2013–2018 in both urban and rural areas, which is consistent with a growing burden of NCD and traffic accidents observed in China (16, 17).

IHD, ranking as the second leading cause of death among Chinese over the observation period (Supplementary Table S5 and Supplementary Figure S1), accounted for a substantial proportion of the gender gap in both areas. The gender gap attributable to IHD increased from 2013–2015 to 2016–2018, especially in rural areas. Consistently, the gender gap attributable to IHD increased in South Korea during 1992–2005 (33). However, there has been a reverse trend in most high-income Western societies, where men have experienced a greater IHD mortality decrease than women over recent decades (3, 4).

China is probably at an earlier stage of the transition in mortality from CVD than most developed countries. A steady decrease in the mortality of CVD including IHD and cerebrovascular disease over past decades in the West suggests that these countries have stepped into a new stage of the mortality transition (8, 9). However, in China, the mortality of IHD has increased over recent years, although the mortality of cerebrovascular disease has decreased (41). As men have a more pronounced increase in the IHD mortality than women (41), it is of no surprise that the gender gap attributable to IHD increased over the observation period.

The increasing gender gap attributable to IHD may be partly due to an emerging obesity epidemic in China (16, 18). On the one hand, women may generally have a lower level of exposure to unhealthy eating associated with obesity than men (42, 43). On the other hand, men may have a greater susceptibility to the adverse effects of the emerging obesity epidemic due to the moderating effects of sex steroids on cardiovascular outcomes (44, 45). However, the gender differences in the levels of exposure and susceptibility to the obesity epidemic and their roles in determining the gender gap remain to be tested in future studies.

Lung cancer, one of the most common cancers among Chinese people (Supplementary Table S5 and Supplementary Figure S1), also accounted for a substantial portion of the gender gap. Furthermore, lung cancer increased the gender gap from 2013–2015 to 2016–2018, especially in rural areas, which was contrary to the declining gender gap attributable to lung cancer over recent decades observed in Western developed countries (3, 4). But the upward trend observed in our study is consistent with the results obtained from Shanghai during 1999–2018 (20) and South Korea during 1992–2005 (33). However, Le et al. (19) showed that the contribution of lung cancer to the gender gap had ups and downs in Chinese cities during 2005–2010. One possible explanation for the inconsistent findings between (19) and ours is the different observation periods. Furthermore, we used data from the national mortality surveillance system that covered 31 provinces, while Le et al. (19) based their analyses on data from dozens of Chinese cities (19).

In many Western developed nations, a convergence in cigarette smoking and smoking-attributed mortality (e.g., lung cancer and IHD mortality) between men and women explains a substantial portion of the decrease in the gender gap that began in the early 1980s (3, 11–13). In China, however, gender differences in cigarette smoking have been increased (10, 46, 47). Under the influences of Confucian patriarchal values, the female smoking prevalence remains low and has been decreased across successive birth cohorts (10, 46, 47). Unfavorably, the male smoking prevalence remains high and has had no notable decrease across cohorts (10, 46, 47). Furthermore, younger male cohorts have initiated cigarette smoking at an earlier age and consumed more cigarettes per smoker than older male cohorts, indicating greater future hazards of cigarette smoking for Chinese men (10, 47). As cigarette smoking has long been recognized as the leading risk factor for deaths from lung cancer and IHD (10), the rising gender differences in the levels of exposure to smoking in the cohort succession (10, 47) may explain why lung cancer and IHD increased the gender gap in both areas.

China has not yet seen the full hazards of cigarette smoking on mortality (10). The contribution of smoking-attributed mortality to the gender gap remains to be investigated in the future when the smoking epidemic steps into a more advanced stage. Furthermore, whether the gender differences in smoking will be narrowed in China (48), as it has been narrowed in Western developed nations, and result in a decrease in the gender gap attributable to smoking-related mortality decades later also requires to be investigated in future studies.

Traffic accidents also contributed a notable portion of the gender gap over the observation period in both urban and rural areas. From 2013–2015 to 2016–2018, this cause of death made the greatest contribution to reducing the gender gap. The narrowing effect of traffic accidents on the gender gap is consistent with the findings from Canada for 1970–2000 and South Korea for 1992–2005. The decreasing gender gap attributable to traffic accidents may be due to improvements in transport regulations, traffic infrastructure, trauma rescue, and hospital treatment (49, 50). These improvements may have led to a more pronounced decrease in the mortality from traffic accidents among men than among women, as the former generally drive more kilometers, are more likely to have unsafe driving, and have a higher initial traffic accident mortality rate than the latter (50).

Our estimates indicate that the gender gap may be smaller in urban areas than in rural areas. Furthermore, there was a notable decrease in the gender gap from 2013–2015 to 2016–2018 in urban areas, which is consistent with the decreasing gender gap observed in most developed societies (3–7). However, the gender gap remained rather constant in rural areas. Lung cancer, IHD, cerebrovascular disease, and chronic lower respiratory disease were responsible for the different trends of the gender gap observed in urban and rural areas.

Rural areas had a more pronounced increase in the gender gap due to lung cancer and IHD than urban areas. One speculative explanation is the urban-rural differences in the smoking epidemic (10). Although the smoking epidemic started earlier in urban than in rural areas, rural men have caught up with urban men in cigarette smoking and had a higher smoking prevalence at least since the mid-1980s (23). Another possible explanation is the urban-rural differences in the obesity epidemic, as obesity is a crucial risk factor of getting IHD (16). Although the obesity epidemic started in urban areas, rural residents have been catching up to their urban counterparts in body mass index in recent decades (24). This might further explain why the increase in the gender gap due to IHD may be more pronounced in rural areas than in urban areas.

Additionally, the gender gap attributable either to cerebrovascular disease or chronic lower respiratory disease decreased in urban areas but increased in rural areas over the observation period. The different findings from urban and rural areas may due to the unequal health care access between urban and rural areas. Rural people generally lag behind their urban peers in adopting upgraded surgical treatments and medications for cerebrovascular disease and chronic lower respiratory disease due to the lower availability and affordability of health care resources in rural areas (51). For example, the mortality from obstructive pulmonary disease (COPD), a main type of chronic lower respiratory disease, has remarkably decreased in China since the early 1990s due to improved COPD diagnosis and management (52). But compared with their urban counterparts, rural residents with COPD have poorer awareness of their diagnosis, more severe conditions, are less likely to seek medical advice, and more likely to die from COPD, given that they generally have less access to health care than their urban peers (53). However, more epidemiological studies that consider both gender and urban-rural heterogeneities are needed to advance our understanding of the relationship between health care access and gender differences in mortality.

A rise in the burden of cervical cancer and breast cancer in Chinese women also narrowed the gender gap in both urban and rural areas. The incidence and mortality rates of breast cancer and cervical cancer among Chinese women have increased over the past decades (54–56). Similarly, breast cancer decreased the female advantage in survival in South Korea during 1992–2015 (33). However, cervical cancer increased the gender gap in South Korea (33), which is inconsistent with our findings. In high-income societies, the introduction of publicly funded national human papillomavirus (HPV) vaccination programs and the expansion of early cancer detection have contributed to reducing the burden of female-dominated cancers (57). Unfortunately, both the HPV vaccination rate and cervical and breast screening rates remain low in China (58, 59). Without the implementation of effective interventions, these female-dominated cancers may continue to reduce the female advantage in survival.

It is important to note that this study is subject to several limitations. First, our observation period is 2013–2018, which is a relatively short period. Further studies are needed to examine whether the decrease in the gender gap in urban areas will continue and whether the gender gap in rural areas will start to decrease after 2018. Second, we are not able to provide uncertainty estimates as the comprehensive data on the number of people and deaths from the national mortality surveillance system is not publicly available. Third, prior evidence showed that Arriaga's method might underestimate the importance of causes of death that are concentrated mainly at older ages (60). Thus, our estimates regarding the contribution of NCD like IHD, cerebrovascular disease, lung cancer, and chronic lower respiratory disease to the gender gap may be conservative. However, the limitation may be less relevant in China than it has been in Western developed countries. Because China is at an earlier stage of mortality transition than Western developed countries. Thus, NCD may account for a smaller proportion of deaths in China than in Western developed countries. Fourth, the national mortality surveillance system is subject to the under-reporting of deaths (61). We do not correct for the under-reporting bias as our estimates of the gender gap would not be biased if the under-reporting of deaths does not vary systematically by gender. Prior evidence suggested that the gender difference in the under-reporting rate of mortality is statistically insignificant in all age groups except for the 0–4 age group (61), which suggests that we may overestimate the gender gap in the 0–4 age groups. However, the 0–4 age group accounted for <3% of the gender gap in both areas during 2013–2018. The mortality rate from IHD, cerebrovascular disease, lung cancer, and chronic lower respiratory disease, the main contributors to the gender gap, was very low in this age group. Therefore, we believe that the effect of the under-reporting bias on our main findings is negligible.

## Conclusion

The gender gap decreased in urban areas but remained rather constant in rural areas from 2013–2015 to 2016–2018. CVD, cancer, respiratory disease, and external causes were the main contributors to the gender gap during 2013–2018. Traffic accidents, among external causes, contributed to most of the decrease in the gender gap from 2013–2015 to 2016–2018 in both urban and rural areas. However, IHD and lung cancer increased the gender gap in both areas, which mitigated the gender gap reduction to a large extent. Furthermore, the gender gap attributable either to cerebrovascular disease or to chronic lower respiratory disease decreased in urban areas but increased in rural areas, suggesting the necessity of implementing urban-rural-specific interventions to improve population health and health equity.

## Data Availability Statement

Publicly available datasets were analyzed in this study. This data can be found at: https://data.cnki.net/yearbook/Single/N2021020144.

## Ethics Statement

Ethical review and approval was not required for the study on human participants in accordance with the local legislation and institutional requirements. Written informed consent from the participants' legal guardian/next of kin was not required to participate in this study in accordance with the national legislation and the institutional requirements.

## Author Contributions

JW and SK conceived the original idea and performed the statistical analysis. ML was invited for his important contributions regarding the contribution of smoking-attributable mortality to the urban/rural specific evolution of the gender gap in life expectancy. JW wrote the first draft of the manuscript. SK and ML revised the manuscript critically for important intellectual contents. All authors contributed to the interpretation of the results. All authors have read and approved the final version of the manuscript.

## Funding

This research was supported by the Shanghai Social Science Foundation (2020ESH003). The funding source has no role in study design, in the collection, analysis and interpretation of data, in the writing of the paper, or in the decision to submit the paper for publication.

## Conflict of Interest

The authors declare that the research was conducted in the absence of any commercial or financial relationships that could be construed as a potential conflict of interest.

## Publisher's Note

All claims expressed in this article are solely those of the authors and do not necessarily represent those of their affiliated organizations, or those of the publisher, the editors and the reviewers. Any product that may be evaluated in this article, or claim that may be made by its manufacturer, is not guaranteed or endorsed by the publisher.
