# Supplementary material for: The Gender Gap in Life Expectancy in Urban and Rural China, 2013–2018
Source: Front Public Health. 2022 Feb 11;10:749238. doi: 10.3389/fpubh.2022.749238 (PMC8873095; doi:10.3389/fpubh.2022.749238)
Supplement: Supplementary file 1 [file Data_Sheet_1.docx]

**SUPPLEMENTARY MATERIAL**

**The gender gap in life expectancy in urban and rural China, 2013-2018**

**Jinjing Wu^1*^, Samir KC^1,2^, Marc Luy^3^**

^1^Asian Demographic Research Institute, Shanghai University, 200444 Shanghai, China.

^2^International Institute for Applied Systems Analysis (IIASA), Wittgenstein Centre for Demography and Global Human Capital (IIASA, OeAW, University of Vienna), 2361 Laxenburg, Austria.

^3^Vienna Institute of Demography (OeAW), Wittgenstein Centre for Demography and Global Human Capital (IIASA, OeAW, University of Vienna), 1030 Vienna, Austria.

***Correspondence:**

Jinjing Wu

E-mail: [jinjingwu1105@gmail.com](mailto:kcsamir@gmail.com,)

**TABLE S1** Life expectancy at birth during 2013-2018

|  | Urban areas | | | Rural areas | | |
| --- | --- | --- | --- | --- | --- | --- |
|  | 2013-2018 | 2013-2015 | 2016-2018 | 2013-2018 | 2013-2015 | 2016-2018 |
| Life expectancy at birth among men, years ^1^ | 77.02 | 76.67 | 77.39 | 75.71 | 75.23 | 76.21 |
| Life expectancy at birth among women, years ^1^ | 82.10 | 81.84 | 82.36 | 81.38 | 80.91 | 81.86 |
| Gender gap in life expectancy at birth, years | 5.08 | 5.17 | 4.98 | 5.66 | 5.68 | 5.65 |

^1^ The life expectancy at birth may be overestimated because the national mortality surveillance system is subject to the under-reporting of deaths. We did not correct for the under-reporting bias as our estimates of the gender gap would not be biased if the under-reporting of deaths did not vary systematically with gender.

**TABLE S2** Age-specific contributions to the gender gap in life expectancy at birth by urban/rural residence during 2013-2018

|  | Urban areas | | Rural areas | |
| --- | --- | --- | --- | --- |
| Age | Year | % | Year | % |
| 0-1 | 0.09 | 1.81 | 0.07 | 1.22 |
| 1-4 | 0.03 | 0.54 | 0.03 | 0.57 |
| 5-9 | 0.02 | 0.44 | 0.03 | 0.62 |
| 10-14 | 0.03 | 0.62 | 0.04 | 0.78 |
| 15-19 | 0.06 | 1.11 | 0.09 | 1.57 |
| 19-24 | 0.05 | 1.06 | 0.09 | 1.63 |
| 25-29 | 0.09 | 1.78 | 0.15 | 2.71 |
| 30-34 | 0.11 | 2.12 | 0.20 | 3.54 |
| 35-39 | 0.13 | 2.50 | 0.20 | 3.58 |
| 40-44 | 0.21 | 4.09 | 0.27 | 4.78 |
| 45-49 | 0.24 | 4.81 | 0.33 | 5.88 |
| 50-54 | 0.48 | 9.41 | 0.52 | 9.12 |
| 55-59 | 0.47 | 9.19 | 0.46 | 8.06 |
| 60-64 | 0.66 | 13.07 | 0.62 | 10.88 |
| 65-69 | 0.70 | 13.84 | 0.69 | 12.10 |
| 70-74 | 0.62 | 12.12 | 0.61 | 10.75 |
| 75-79 | 0.46 | 9.10 | 0.54 | 9.62 |
| 80-84 | 0.33 | 6.60 | 0.40 | 6.98 |
| 85+ | 0.30 | 5.81 | 0.32 | 5.63 |
| Total | 5.08 | 100 | 5.66 | 100 |

**Table S3** Age-specific contribution to the change in life expectancy at birth, gender gap in life expectancy at birth, and changing gender gap in life expectancy in urban areas

| Age | Life expectancy at birth change from  2013-2015 to 2016-2018 | | | | Gender gap in life expectancy at birth | | | | Gender gap change from 2013-2015 to 2016-2018 | |
| --- | --- | --- | --- | --- | --- | --- | --- | --- | --- | --- |
|  | Male |  | Female |  | 2013-2015 | | 2016-2018 | |  |  |
|  | Years | % | Years | % | Years | % | Years | % | Years | % |
| 0-1 | 0.11 | 15.75 | 0.08 | 14.81 | 0.11 | 2.18 | 0.07 | 1.42 | -0.04 | 21.65 |
| 1-4 | 0.03 | 4.69 | 0.03 | 5.56 | 0.03 | 0.6 | 0.02 | 0.49 | -0.01 | 3.43 |
| 5-9 | 0.01 | 1.78 | 0.01 | 1.07 | 0.03 | 0.51 | 0.02 | 0.37 | -0.01 | 4.07 |
| 10-14 | 0.01 | 1.9 | 0.01 | 1.33 | 0.04 | 0.68 | 0.03 | 0.55 | -0.01 | 3.83 |
| 15-19 | 0.03 | 3.89 | 0.01 | 1.56 | 0.07 | 1.29 | 0.05 | 0.91 | -0.02 | 11.03 |
| 19-24 | 0.04 | 5.08 | 0.02 | 2.95 | 0.07 | 1.27 | 0.04 | 0.84 | -0.02 | 12.19 |
| 25-29 | 0.02 | 2.7 | 0.01 | 2.69 | 0.09 | 1.81 | 0.09 | 1.75 | -0.01 | 3.2 |
| 30-34 | 0.01 | 1.34 | 0.01 | 1.78 | 0.11 | 2.08 | 0.11 | 2.16 | 0 | 0.07 |
| 35-39 | 0.03 | 4.74 | 0.01 | 2.69 | 0.14 | 2.67 | 0.12 | 2.32 | -0.02 | 11.47 |
| 40-44 | 0.09 | 12.66 | 0.04 | 8.56 | 0.23 | 4.54 | 0.18 | 3.61 | -0.06 | 28.23 |
| 45-49 | 0.03 | 4.51 | 0.01 | 2.07 | 0.26 | 4.93 | 0.23 | 4.68 | -0.02 | 11.28 |
| 50-54 | -0.06 | -8.75 | -0.05 | -8.8 | 0.46 | 8.89 | 0.5 | 9.96 | 0.04 | -18.39 |
| 55-59 | 0.11 | 14.69 | 0.07 | 13.7 | 0.49 | 9.44 | 0.44 | 8.92 | -0.04 | 22.74 |
| 60-64 | -0.1 | -13.98 | -0.03 | -4.8 | 0.61 | 11.79 | 0.72 | 14.42 | 0.11 | -55.42 |
| 65-69 | -0.02 | -3.33 | 0.02 | 3.81 | 0.67 | 13 | 0.73 | 14.72 | 0.06 | -30.74 |
| 70-74 | 0.15 | 20.91 | 0.15 | 28.36 | 0.63 | 12.2 | 0.6 | 12.03 | -0.03 | 16.36 |
| 75-79 | 0.23 | 31.83 | 0.26 | 49.13 | 0.48 | 9.36 | 0.44 | 8.81 | -0.05 | 23.47 |
| 80-84 | 0.1 | 14.34 | 0.08 | 15.67 | 0.36 | 6.97 | 0.31 | 6.2 | -0.05 | 26.46 |
| 85+ | -0.11 | -14.75 | -0.22 | -42.14 | 0.3 | 5.81 | 0.29 | 5.84 | -0.01 | 5.08 |
| Total | 0.71 | 100 | 0.52 | 100 | 5.18 | 100 | 4.99 | 100 | -0.19 | 100 |

**Table S4** Age-specific contribution to the change in life expectancy at birth, gender gap in life expectancy at birth, and changing gender gap in life expectancy in rural areas

| Age | Life expectancy at birth change from  2013-2015 to 2016-2018 | | | | Gender gap in life expectancy at birth | | | | Gender gap change from 2013-2015 to 2016-2018 | |
| --- | --- | --- | --- | --- | --- | --- | --- | --- | --- | --- |
|  | Male |  | Female |  | 2013-2015 | | 2016-2018 | |  |  |
|  | Years | % | Years | % | Years | % | Years | % | Years | % |
| 0-1 | 0.1 | 10.35 | 0.09 | 9.95 | 0.08 | 1.33 | 0.06 | 1.1 | -0.01 | 51.97 |
| 1-4 | 0.04 | 3.81 | 0.03 | 3.01 | 0.04 | 0.67 | 0.03 | 0.47 | -0.01 | 44.12 |
| 5-9 | 0.02 | 1.93 | 0.01 | 1.06 | 0.04 | 0.7 | 0.03 | 0.53 | -0.01 | 39.19 |
| 10-14 | 0.01 | 0.99 | 0.01 | 0.59 | 0.05 | 0.81 | 0.04 | 0.74 | 0 | 17.36 |
| 15-19 | 0.03 | 3.41 | 0.01 | 0.9 | 0.1 | 1.8 | 0.08 | 1.34 | -0.03 | 106.41 |
| 19-24 | 0.05 | 4.84 | 0.02 | 2.22 | 0.11 | 1.88 | 0.08 | 1.37 | -0.03 | 117.73 |
| 25-29 | 0.03 | 2.95 | 0.01 | 1.19 | 0.16 | 2.86 | 0.14 | 2.55 | -0.02 | 71.41 |
| 30-34 | 0.02 | 1.68 | 0.01 | 0.86 | 0.2 | 3.58 | 0.2 | 3.49 | -0.01 | 24.55 |
| 35-39 | 0.04 | 3.77 | 0.02 | 2.4 | 0.21 | 3.7 | 0.2 | 3.46 | -0.01 | 57.97 |
| 40-44 | 0.08 | 8.44 | 0.04 | 4.45 | 0.29 | 5.17 | 0.25 | 4.39 | -0.05 | 181.03 |
| 45-49 | 0.02 | 1.67 | 0.01 | 1.16 | 0.33 | 5.85 | 0.33 | 5.91 | 0 | -7.17 |
| 50-54 | -0.16 | -16.8 | -0.09 | -9.15 | 0.46 | 8.05 | 0.58 | 10.23 | 0.12 | -487.37 |
| 55-59 | 0.11 | 11.33 | 0.07 | 7.62 | 0.48 | 8.44 | 0.43 | 7.68 | -0.04 | 180.11 |
| 60-64 | -0.04 | -4.33 | -0.01 | -0.59 | 0.58 | 10.26 | 0.65 | 11.54 | 0.07 | -281.55 |
| 65-69 | 0.07 | 7.22 | 0.08 | 8.34 | 0.68 | 11.89 | 0.7 | 12.31 | 0.02 | -83.24 |
| 70-74 | 0.17 | 17.54 | 0.15 | 15.25 | 0.63 | 11.07 | 0.59 | 10.41 | -0.04 | 162.08 |
| 75-79 | 0.22 | 22.85 | 0.24 | 25.52 | 0.56 | 9.82 | 0.53 | 9.38 | -0.03 | 109.09 |
| 80-84 | 0.15 | 15.63 | 0.19 | 19.78 | 0.4 | 6.97 | 0.39 | 6.96 | 0 | 8.87 |
| 85+ | 0.03 | 2.73 | 0.05 | 5.44 | 0.29 | 5.17 | 0.35 | 6.13 | 0.05 | -212.56 |
| Total | 0.99 | 100 | 0.94 | 100 | 5.69 | 100 | 5.66 | 100 | -0.03 | 100 |

**Table S5** Cause-specific contribution to the change in life expectancy at birth, gender gap in life expectancy at birth, and changing gender gap in life expectancy in urban areas

| Causes of death | Life expectancy at birth change from  2013-2015 to 2016-2018 | | | | Gender gap in life expectancy at birth | | | | Gender gap change from 2013-2015 to 2016-2018 | |
| --- | --- | --- | --- | --- | --- | --- | --- | --- | --- | --- |
|  | Male | | Female | | 2013-2015 | | 2016-2018 | |  |  |
|  | Years | % | Years | % | Years | % | Years | % | Years | % |
| **Cancers** | 0.12 | 17.14 | 0.08 | 14.42 | 1.73 | 33.44 | 1.7 | 34.05 | -0.03 | 17.78 |
| Malignant tumors | 0.12 | 17.08 | 0.07 | 13.95 | 1.73 | 33.37 | 1.69 | 33.95 | -0.04 | 18.47 |
| Nasopharyngeal cancer | 0 | 0.52 | 0 | 0.18 | 0.03 | 0.55 | 0.03 | 0.51 | 0 | 1.43 |
| Esophagus cancer | 0.01 | 1.15 | 0 | 0.49 | 0.2 | 3.83 | 0.2 | 3.95 | 0 | 0.76 |
| Stomach cancer | 0.03 | 4.21 | 0.02 | 4.35 | 0.25 | 4.93 | 0.25 | 4.94 | -0.01 | 4.58 |
| Colorectal cancer | -0.01 | -1.06 | 0 | 0.18 | 0.09 | 1.65 | 0.1 | 1.95 | 0.01 | -6.21 |
| Liver cancer | 0.03 | 4.75 | 0.01 | 2.75 | 0.43 | 8.25 | 0.41 | 8.24 | -0.02 | 8.66 |
| Lung cancer | 0.02 | 2.31 | 0.03 | 5.07 | 0.69 | 13.24 | 0.7 | 14.15 | 0.02 | -9.77 |
| Breast cancer | 0 | 0.05 | -0.01 | -1.74 | -0.15 | -3 | -0.17 | -3.35 | -0.01 | 5.99 |
| Cervical cancer | 0 | 0 | -0.01 | -2.28 | -0.08 | -1.56 | -0.09 | -1.88 | -0.01 | 6.43 |
| Bladder cancer | 0 | 0 | 0 | 0.03 | 0.04 | 0.76 | 0.04 | 0.8 | 0 | -0.45 |
| Leukocythemia | 0.01 | 1.19 | 0.01 | 1.73 | 0.03 | 0.51 | 0.03 | 0.53 | 0 | -0.08 |
| **Cardiovascular diseases** | 0.15 | 20.85 | 0.12 | 23.43 | 1.61 | 31.06 | 1.61 | 32.38 | 0 | -2.5 |
| Heart disease | 0.02 | 3.09 | 0 | 0.38 | 0.69 | 13.32 | 0.71 | 14.2 | 0.02 | -9.15 |
| Chronic rheumatic  heart disease | 0 | 0.59 | 0.01 | 1.92 | -0.01 | -0.23 | -0.01 | -0.15 | 0 | -2.19 |
| Hypertensive  cardiopathy | 0 | 0.03 | -0.02 | -3.55 | 0.05 | 0.94 | 0.04 | 0.82 | -0.01 | 3.88 |
| Ischemic heart disease | -0.06 | -7.84 | -0.06 | -11.89 | 0.54 | 10.45 | 0.59 | 11.88 | 0.05 | -26.1 |
| Cerebrovascular  disease | 0.11 | 15.83 | 0.1 | 18.42 | 0.86 | 16.7 | 0.85 | 17.04 | -0.02 | 8.11 |
| Other hypertensive  disease | 0.02 | 3.12 | 0.03 | 5.86 | 0.03 | 0.65 | 0.03 | 0.62 | 0 | 1.44 |
| **External causes** | 0.15 | 21.19 | 0.04 | 7.59 | 0.69 | 13.3 | 0.58 | 11.68 | -0.11 | 54.61 |
| Traffic accidents | 0.09 | 13.11 | 0.03 | 5.05 | 0.32 | 6.15 | 0.25 | 4.92 | -0.07 | 37.47 |
| Accidental fall | -0.01 | -1.61 | -0.03 | -4.82 | 0.1 | 1.92 | 0.1 | 1.99 | 0 | 0.33 |
| Drawn | 0.02 | 3.1 | 0.01 | 2.49 | 0.07 | 1.35 | 0.06 | 1.2 | -0.01 | 5.18 |
| Suicide | 0.01 | 1.64 | 0.02 | 2.99 | 0.04 | 0.7 | 0.04 | 0.76 | 0 | -0.88 |
| **Respiratory disease** | 0.18 | 24.97 | 0.19 | 35.48 | 0.63 | 12.13 | 0.58 | 11.59 | -0.05 | 25.71 |
| Pneumonia | 0.03 | 4.25 | 0.03 | 4.83 | 0.11 | 2.21 | 0.11 | 2.14 | -0.01 | 4 |
| Chronic lower respiratory disease | 0.13 | 17.38 | 0.14 | 26.28 | 0.44 | 8.54 | 0.41 | 8.24 | -0.03 | 16.15 |
| **Digestive disease** | 0.01 | 2.02 | 0.02 | 3.74 | 0.16 | 3.18 | 0.17 | 3.42 | 0.01 | -2.98 |
| Gastric and duodenal ulcer | 0 | 0.33 | 0 | 0.27 | 0.02 | 0.44 | 0.02 | 0.45 | 0 | 0.34 |
| Intestinal obstruction | 0 | 0.18 | 0 | 0.34 | 0.01 | 0.13 | 0.01 | 0.14 | 0 | -0.09 |
| Liver disease | 0.01 | 1.8 | 0.01 | 1.28 | 0.11 | 2.08 | 0.1 | 2.03 | -0.01 | 3.2 |
| **Infectious disease** | 0.02 | 3.13 | 0.02 | 3.51 | 0.11 | 2.18 | 0.11 | 2.16 | -0.01 | 2.93 |
| Tuberculosis | 0 | 0.63 | 0 | 0.47 | 0.04 | 0.69 | 0.03 | 0.67 | 0 | 1.1 |
| Hepatitis | 0 | 0.46 | 0 | 0.62 | 0.04 | 0.85 | 0.04 | 0.89 | 0 | -0.11 |
| AIDS | 0 | 0.02 | 0 | 0.05 | 0.01 | 0.27 | 0.01 | 0.29 | 0 | -0.23 |
| **Endocrine, nutritional & metabolic disease** | -0.03 | -3.99 | -0.02 | -4.12 | 0.02 | 0.46 | 0.04 | 0.88 | 0.02 | -10.46 |
| Diabetes | -0.03 | -4.01 | -0.02 | -3.21 | 0.02 | 0.35 | 0.04 | 0.8 | 0.02 | -11.25 |
| **Unspecified causes of death** | 0 | 0.09 | 0 | 0.8 | 0.04 | 0.73 | 0.04 | 0.8 | 0 | -0.89 |
| **Others ^1^** | 0.11 | 14.6 | 0.08 | 15.14 | 0.18 | 3.53 | 0.15 | 3.04 | -0.03 | 15.8 |
| **Total** | 0.71 | 100.00 | 0.53 | 99.99 | 5.17 | 100.01 | 4.98 | 100.00 | -0.20 | 100.00 |

^1^ Other causes include 1) diseases of the blood and blood-forming organs and certain disorders involving the immune mechanism, 2) mental, behavioral and neurodevelopmental disorders, 3) diseases of the nervous system, 4) diseases of the musculoskeletal system and connective tissue, 5) diseases of the genitourinary system, pregnancy, childbirth and the puerperium, 6) certain conditions originating in the perinatal period, and 7) congenital malformations, deformations and chromosomal abnormalities.

**Table S6** Cause-specific contribution to the change in life expectancy at birth, gender gap in life expectancy at birth, and changing gender gap in life expectancy in rural areas

| Causes of death | Life expectancy at birth change from  2013-2015 to 2016-2018 | | | | Gender gap in life expectancy at birth | | | | Gender gap change from 2013-2015 to 2016-2018 | |
| --- | --- | --- | --- | --- | --- | --- | --- | --- | --- | --- |
|  | Male |  | Female |  | 2013-2015 | | 2016-2018 | |  |  |
|  | Years | % | Years | % | Years | % | Years | % | Years | % |
| **Cancers** | 0.13 | 12.98 | 0.07 | 7.09 | 1.71 | 30.21 | 1.73 | 30.54 | 0.01 | -45.52 |
| Malignant tumors | 0.13 | 13.03 | 0.07 | 6.95 | 1.71 | 30.17 | 1.72 | 30.47 | 0.01 | -37.54 |
| Nasopharyngeal cancer | 0 | -0.22 | 0 | -0.09 | 0.03 | 0.5 | 0.03 | 0.56 | 0 | -11.84 |
| Esophagus cancer | 0.03 | 2.87 | 0.02 | 2.6 | 0.22 | 3.94 | 0.22 | 3.98 | 0 | -5.27 |
| Stomach cancer | 0.05 | 5.07 | 0.04 | 3.68 | 0.29 | 5.12 | 0.28 | 4.93 | -0.01 | 47.29 |
| Colorectal cancer | -0.01 | -1.42 | -0.01 | -1.02 | 0.06 | 1.01 | 0.07 | 1.24 | 0.01 | -51.11 |
| Liver cancer | 0.03 | 3.13 | 0.02 | 2.17 | 0.49 | 8.58 | 0.49 | 8.66 | 0 | -11.17 |
| Lung cancer | 0 | -0.33 | 0 | 0.45 | 0.56 | 9.87 | 0.61 | 10.72 | 0.05 | -184.5 |
| Breast cancer | 0 | 0.01 | -0.01 | -1.4 | -0.11 | -1.93 | -0.13 | -2.24 | -0.02 | 68.58 |
| Cervical cancer | 0 | 0 | -0.02 | -2.6 | -0.08 | -1.42 | -0.11 | -1.89 | -0.03 | 105.72 |
| Bladder cancer | 0 | -0.1 | 0 | -0.08 | 0.03 | 0.51 | 0.03 | 0.58 | 0 | -15.25 |
| Leukocythemia | 0 | 0.18 | 0 | 0.51 | 0.03 | 0.45 | 0.03 | 0.53 | 0 | -17.78 |
| **Cardiovascular diseases** | 0.35 | 35.79 | 0.45 | 47.08 | 1.79 | 31.52 | 1.84 | 32.5 | 0.05 | -191.67 |
| Heart disease | 0.1 | 10.02 | 0.15 | 15.88 | 0.72 | 12.63 | 0.76 | 13.45 | 0.04 | -173.84 |
| Chronic rheumatic  heart disease | 0.01 | 0.61 | 0.01 | 1.23 | -0.01 | -0.18 | -0.01 | -0.14 | 0 | -10.5 |
| Hypertensive  cardiopathy | 0.02 | 2.51 | 0.03 | 2.69 | 0.06 | 1.1 | 0.06 | 1.03 | 0 | 16.17 |
| Ischemic heart disease | -0.06 | -5.68 | -0.04 | -3.91 | 0.55 | 9.73 | 0.63 | 11.23 | 0.08 | -331.15 |
| Cerebrovascular  disease | 0.19 | 19.14 | 0.22 | 23.52 | 1.01 | 17.79 | 1.03 | 18.28 | 0.02 | -94.26 |
| Other hypertensive  disease | 0.07 | 6.82 | 0.07 | 7.43 | 0.05 | 0.84 | 0.02 | 0.4 | -0.03 | 100.98 |
| **External causes** | 0.23 | 23.24 | 0.08 | 8.76 | 1.12 | 19.76 | 0.99 | 17.54 | -0.13 | 523.47 |
| Traffic accidents | 0.13 | 13.61 | 0.03 | 3.22 | 0.53 | 9.42 | 0.43 | 7.66 | -0.1 | 409.32 |
| Accidental fall | -0.02 | -1.55 | -0.02 | -2.41 | 0.14 | 2.46 | 0.15 | 2.64 | 0.01 | -37.29 |
| Drawn | 0.03 | 3.32 | 0.02 | 1.81 | 0.12 | 2.04 | 0.1 | 1.76 | -0.02 | 65.41 |
| Suicide | 0.02 | 1.9 | 0.03 | 3.43 | 0.06 | 1 | 0.07 | 1.17 | 0.01 | -38.34 |
| **Respiratory disease** | 0.14 | 14.34 | 0.22 | 23.13 | 0.54 | 9.43 | 0.59 | 10.37 | 0.05 | -204.45 |
| Pneumonia | 0.03 | 2.64 | 0.03 | 3.56 | 0.05 | 0.95 | 0.06 | 1.02 | 0 | -13.01 |
| Chronic lower respiratory disease | 0.11 | 11.36 | 0.17 | 18.22 | 0.44 | 7.68 | 0.47 | 8.39 | 0.04 | -153.05 |
| **Digestive disease** | 0.03 | 2.81 | 0.02 | 2.61 | 0.19 | 3.41 | 0.19 | 3.43 | 0 | -1.32 |
| Gastric and duodenal ulcer | 0.01 | 0.89 | 0.01 | 0.58 | 0.03 | 0.55 | 0.03 | 0.49 | 0 | 14.73 |
| Intestinal obstruction | 0 | 0.08 | 0 | 0.13 | 0.01 | 0.1 | 0.01 | 0.11 | 0 | -2.34 |
| Liver disease | 0.01 | 1.38 | 0.01 | 1.19 | 0.12 | 2.15 | 0.12 | 2.14 | 0 | 4.93 |
| **Infectious disease** | 0.02 | 2.22 | 0.02 | 1.93 | 0.13 | 2.22 | 0.12 | 2.18 | 0 | 9.97 |
| Tuberculosis | 0.01 | 0.66 | 0 | 0.38 | 0.04 | 0.74 | 0.04 | 0.71 | 0 | 8.38 |
| Hepatitis | 0 | 0.25 | 0 | 0.31 | 0.05 | 0.88 | 0.05 | 0.92 | 0 | -6.67 |
| AIDS | 0 | -0.42 | 0 | -0.08 | 0.01 | 0.2 | 0.02 | 0.27 | 0 | -17.15 |
| **Endocrine, nutritional & metabolic disease** | -0.02 | -2.5 | -0.03 | -2.88 | -0.01 | -0.25 | -0.01 | -0.09 | 0.01 | -36.87 |
| Diabetes | -0.02 | -2.07 | -0.02 | -1.96 | -0.02 | -0.3 | -0.01 | -0.14 | 0.01 | -36.62 |
| **Unspecified causes of death** | -0.02 | -2.22 | 0.01 | 1.18 | 0.03 | 0.45 | 0.06 | 1.09 | 0.04 | -144.57 |
| **Others ^1^** | 0.13 | 13.34 | 0.11 | 11.09 | 0.19 | 3.26 | 0.14 | 2.43 | -0.05 | 190.96 |
| **Total** | 0.99 | 100 | 0.95 | 99.99 | 5.69 | 100.01 | 5.65 | 99.99 | -0.02 | 100 |

^1^ Other causes include 1) diseases of the blood and blood-forming organs and certain disorders involving the immune mechanism, 2) mental, behavioral and neurodevelopmental disorders, 3) diseases of the nervous system, 4) diseases of the musculoskeletal system and connective tissue, 5) diseases of the genitourinary system, pregnancy, childbirth and the puerperium, 6) certain conditions originating in the perinatal period, and 7) congenital malformations, deformations and chromosomal abnormalities.

**TABLE S7** Age-standardized cause-specific mortality rates during 2013-2018 by gender and urban/rural residence ^1^

|  | Urban areas | | Rural areas | |
| --- | --- | --- | --- | --- |
|  | Male, per 100,000 people | Female, per 100,000 people | Male, per 100,000 people | Female, per 100,000 people |
| **ICD10** |  |  |  |  |
| **Total** | 631.74 | 391.82 | 698.10 | 424.55 |
| **Cancer** | 180.10 | 94.96 | 176.58 | 88.93 |
| Malignant tumors | 178.61 | 93.72 | 175.48 | 87.98 |
| Nasopharyngeal cancer | 1.85 | 0.60 | 2.15 | 0.72 |
| Esophagus cancer | 13.97 | 4.22 | 16.97 | 5.42 |
| Stomach cancer | 21.97 | 9.29 | 25.44 | 10.60 |
| Colorectal cancer | 12.69 | 8.06 | 8.97 | 5.65 |
| Liver cancer | 29.28 | 9.75 | 34.77 | 11.38 |
| Lung cancer | 57.87 | 23.06 | 50.73 | 20.54 |
| Breast cancer | 0.14 | 7.67 | 0.14 | 5.67 |
| Cervical cancer | 0.00 | 4.02 | 0.00 | 4.47 |
| Bladder cancer | 2.90 | 0.75 | 2.22 | 0.51 |
| Leukocythemia | 3.79 | 2.71 | 3.92 | 2.82 |
| **Cardiovascular disease** | 259.69 | 181.82 | 302.07 | 209.35 |
| Heart disease | 127.58 | 94.83 | 139.93 | 103.12 |
| Chronic rheumatic heart diseases | 2.15 | 2.66 | 2.60 | 3.07 |
| Hypertensive cardiopathy | 11.69 | 9.55 | 16.88 | 13.68 |
| Ischemic heart disease | 102.23 | 75.65 | 106.82 | 77.40 |
| Other hypertension disease | 5.27 | 3.67 | 5.86 | 3.96 |
| Cerebrovascular disease | 123.99 | 81.50 | 153.78 | 100.69 |
| **External cause** | 45.42 | 21.43 | 70.06 | 29.29 |
| Traffic accidents | 17.38 | 6.40 | 28.04 | 9.04 |
| Accidental fall | 9.40 | 5.39 | 11.83 | 5.79 |
| Drawn | 3.29 | 1.65 | 5.30 | 2.55 |
| Suicide | 5.10 | 3.53 | 8.77 | 6.05 |
| **Respiratory disease** | 74.25 | 41.80 | 79.49 | 48.54 |
| Pneumonia | 14.68 | 9.09 | 8.92 | 6.21 |
| Chronic lower respiratory disease | 52.44 | 29.05 | 65.48 | 39.83 |
| **Digestive disease** | 15.81 | 8.19 | 16.95 | 7.72 |
| Gastric and duodenal ulcer | 2.35 | 1.25 | 2.95 | 1.45 |
| Intestinal obstruction | 0.99 | 0.68 | 0.80 | 0.52 |
| Liver disease | 7.38 | 2.60 | 8.12 | 2.39 |
| **Infectious disease** | 8.19 | 3.22 | 9.73 | 3.96 |
| Tuberculosis | 2.20 | 0.55 | 2.96 | 0.94 |
| Hepatitis | 3.34 | 1.32 | 3.94 | 1.57 |
| AIDS | 0.77 | 0.18 | 0.78 | 0.21 |
| **Endocrine, nutritional & metabolic disease** | 16.30 | 14.98 | 11.69 | 12.38 |
| Diabetes | 14.25 | 13.07 | 9.98 | 10.77 |
| **Others** ^2^ | 29.08 | 24.13 | 28.29 | 23.01 |
| **Unspecified causes of death** | 2.90 | 1.28 | 3.25 | 1.37 |

^1^ The 2010 China Census of Population is used as the standard population. We used the 2010 population’s age structure to estimate age-standardized cause-specific mortality rates.

^2^ Other causes include 1) diseases of the blood and blood-forming organs and certain disorders involving the immune mechanism, 2) mental, behavioral and neurodevelopmental disorders, 3) diseases of the nervous system, 4) diseases of the musculoskeletal system and connective tissue, 5) diseases of the genitourinary system, pregnancy, childbirth and the puerperium, 6) certain conditions originating in the perinatal period, and 7) congenital malformations, deformations and chromosomal abnormalities.

**
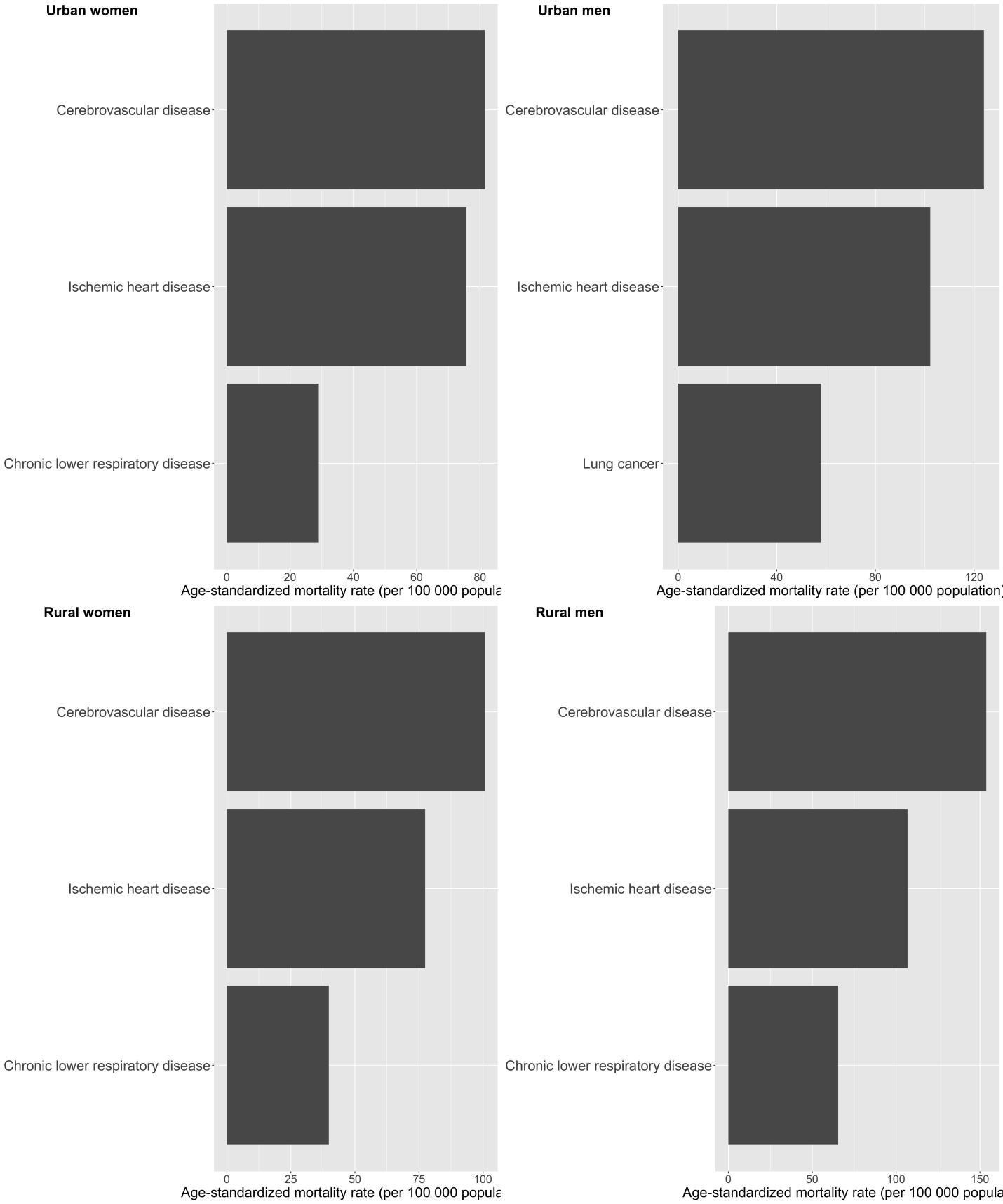
**

**FIGURE S1** Ten leading causes of death by gender and urban/rural residence
